# Special issue: Alternatives to antibiotics in aquaculture – past, present and future

**DOI:** 10.1016/j.cirep.2025.200260

**Published:** 2025-11-04

**Authors:** Margaret Crumlish, Ha Thanh Dong

**Affiliations:** a University of Stirling, Stirling, United Kingdom; b Asian Institute of Technology, Khlong Nueng, Thailand

## Background

Global aquaculture continues to confront significant challenges in disease management. Despite considerable advances in biosecurity, husbandry, and pathogen control, the sector remains heavily reliant on antibiotics, contributing to the emergence of antimicrobial resistance (AMR), treatment failures, environmental contamination, and food safety concerns. As global demand for aquatic products increases, there is an urgent need for sustainable, effective alternatives that enhance fish and shrimp health, mitigate AMR, support environmental sustainability and food security under the One Health framework. This special issue highlights recent research on immunostimulants and vaccination strategies, focusing on innovative approaches that modulate immunity, reduce disease burden, and support resilient and sustainable aquaculture systems.

## Summary of recent publications

This special issue features four peer-reviewed studies that offer new insights into developing alternatives to antibiotics in aquaculture. The study by Fajei et al. [2] provides new insights into the immunomodulatory role of the antimicrobial peptide pituitary adenylate cyclase-activating polypeptide (PACAP). This peptide is naturally found in various organisms, including Nile tilapia (*Oreochromis niloticus*) exposed to *Flavobacterium columnare*. Intraperitoneal administration of peptide PACAP-38 notably reduced mortality and suppressed inflammatory and stress responses in tilapia, as evidenced by lower IL1β expression and cortisol levels. In contrast, a modified PACAP-38 form showed limited protective effects. These findings highlight the promise of PACAP-38 as a potential therapeutic agent to enhance disease resistance and welfare in aquaculture species. Betancourt et al. [1] extend this investigation to juvenile shrimp (*Litopenaeus vannamei*), evaluating PACAP-38 and its modified sequences across different doses and administration routes. The study reveals that PACAP modulates key immune effectors, including hemolymph parameters and gene expression in shrimp hemocytes, gill, and hepatopancreas tissues, in a tissue-and dose-dependent manner. These findings emphasize the potential of PACAP peptides as natural immunostimulants and as promising alternatives to antibiotics for disease prevention in crustaceans.

Vaccination approaches are also highlighted in this issue. Lan et al. [3] demonstrate a non-invasive vaccination strategy against *Vibrio vulnificus* in Asian seabass (*Lates calcarifer*), using an immersion prime model followed by oral boosters. This approach elicited strong systemic, mucosal, and gut-associated immune responses, as evidenced by upregulation of cytokines and T-cell markers, and conferred 84.6 % relative percent survival upon bacterial challenge under experimental conditions. Similarly, Le et al. [4] identify a live attenuated *Vibrio harveyi* strain (VH 2886) as a promising candidate for mucosal vaccination in seabass. Immersion vaccination, particularly when combined with an oral booster, generated high IgM levels, enhanced bactericidal activity, and achieved up to 72 % survival, while safety testing confirmed the strain remained non-pathogenic after multiple passages.

Collectively, these studies emphasis the potential role of immunostimulants and vaccination strategies as viable, sustainable alternatives to unnecessary antibiotics in aquaculture. The studies demonstrate that strategically designed health management interventions can modulate host immunity, enhance disease resistance, promoting better animal and environmental welfare towards disease control. By advancing our understanding of protective immune mechanisms, the research in this special issue builds on a foundational basis for the development of further non-antibiotic innovations in aquaculture health management. As the industry seeks to balance productivity with sustainability while upholding animal welfare, such alternatives, combined with noninvasive effective delivery methods and cost-effectiveness, will be essential for promoting resilient, safe, and responsible aquaculture practices worldwide.

## References

[1] Betancourt J.L., Rodríguez-Ramos T., Rivera L., Carpio Y., Estrada M.P., Ramos L., Dixon B. (2024). Immunomodulatory effects of PACAP sequence modifications in juvenile white shrimp *Litopenaeus vannamei*. Compar. Immunol. Rep..

[2] Fajei E., Cai W.C., Whyte S.K., Despres B., Dixon B., Carpio Y., Estrada M., Fast M.D. (2024). Assessing the impact of two forms of PACAP-38 (pituitary adenylate cyclase-activating polypeptide) on Nile tilapia (*Oreochromis niloticus*) immune response following challenge with *Flavobacterium columnare*. Compar. Immunol. Rep..

[3] Lan N.G.T., Dong H.T., Vinh N.T., Senapin S. (2025). Non-invasive vaccination enhances immune response and protects Asian seabass (*Lates calcarifer*) against *Vibrio vulnificus* infection. Compar. Immunol. Rep..

[4] Le T.T.T., Tossawas P., Panphut W., Sangsuriya P., Phiwsaiya K., Dong H.T., Senapin S. (2025). Evaluation of a live attenuated *Vibrio harveyi* VH 2886 mutant for mucosal vaccination in Asian seabass (*Lates calcarifer*). Compar. Immunol. Rep..

